# Relevant Aspects in the Mechanical and Aging Degradation of NiTi Alloy with R-Phase in Endodontic Files

**DOI:** 10.3390/ma17133351

**Published:** 2024-07-06

**Authors:** Patricia Sánchez, Benedetta Vidi, Cristina Rico, Jesús Mena-Alvarez, Javier Gil, Juan Manuel Aragoneses

**Affiliations:** 1 Bioengineering Institute of Technology, Facultad de Medicina y Ciencias de la Salud, Universitat Internacional de Catalunya, 08195 Sant Cugat del Vallés, Spain; patricia.sanchez@uax.es; 2 Programa de Doctorado Ciencia de la Salud, Universidad de Alcalá, Pza. San Diego s/n, 28801 Alcalá de Henares, Spain; benedetta.vidi@uax.es; 3 Faculty of Dentistry, Universidad Alfonso X El Sabio, C. de Emilio Muñoz, 13, 28691 Madrid, Spain; cromaric@uax.es (C.R.); jaraglam@uax.es (J.M.A.); 4 Department of Dental Research, Federico Henriquez y Carvajal University, Santo Domingo 10106, Dominican Republic

**Keywords:** NiTi, endodontic files, R-phase, superelasticity, fatigue

## Abstract

One of the most important challenges in endodontics is to have files that have excellent flexibility, toughness, and high fatigue life. Superelastic NiTi alloys have been a breakthrough and the new R-phase NiTi alloys promise to further optimize the good properties of NiTi alloys. In this work, two austenitic phase endodontic files with superelastic properties (Protaper and F6) and two austenitic phase files with the R-phase (M-wire and Reciproc) have been studied. The transformation temperatures were studied by calorimetry. Molds reproducing root canals at different angles (30, 45, and 70°) were obtained with cooling and loads simulating those used in the clinic. Mechanical cycles of different files were realized to fracture. Transformation temperatures were determined at different number of cycles. The different files were heat treated at 300 and 500 °C as the aging process, and the transformation temperatures were also determined. Scanning and transmission electron microscopy was used to observe the fractography and precipitates of the files. The results show that files with the R-phase have higher fracture cycles than files with only the austenitic phase. The fracture cycles depend on the angle of insertion in the root canal, with the angle of 70° being the one with the lowest fracture cycles in all cases. The R-Phase transformation increases the energy absorbed by the NiTi to produce the austenitic to R-phase and to produce the martensitic transformation causing the increase in the fracture cycles. Mechanical cycling leads to significant increases in the transformation temperatures M_s_ and A_f_ as well as R_s_ and R_f_. No changes in the transformation temperatures were observed for aging at 300 °C, but the appearance of Ni_4_Ti_3_ precipitates was observed in the aging treatments to the Nickel-rich files that correspond to those with the R transition. These results should be considered by endodontists to optimize the type of files for clinical therapy.

## 1. Introduction

NiTi alloy presents a thermoelastic martensitic transformation and gives rise to shape memory and pseudoelasticity properties that are of interest for their application in different fields of medicine, such as orthodontics, rehabilitation, cardiovascular medicine, and endodontics, among others [1,2,3,4]. This reversible transformation is produced from the matrix phase or austenite B2 (A) and the B19′ (M) phase called martensite.

The chemical compositions of NiTi alloys have been optimized and new thermomechanical treatments have been developed with different heat treatments that allow an intermediate phase in the martensitic transformation to be obtained. This intermediate phase is called the R-phase and was also called the pre-martensitic transition. The R-phase is a rhombohedral structure between B2 and B19′. The transformation is B2 → R → B19′ [5,6].

According to Landau’s theories on the free energy associated with B2 B19′ and B2 → R → B19′, it is suggested that this multiple martensitic transformation takes place by the sequence of an increase in the deformation generated by the transformation or by an increase in the transformation entropy. In fully annealed NiTi alloys, the B19′ phase is more stable than the R-phase and consequently by cooling the B2 → B19′ transformation takes place [7].

The formation of dislocation tangles or the precipitation of coherent Ni_4_Ti_3_ compounds in nickel-rich NiTi alloys causes an obstacle to the martensitic B2 → B19′ transformation, and the R-phase appears [8,9]. The distortion in the formation of the R-phase is about 1%, which is much smaller than that associated with the direct transformation to B19′, which is about 10%; this makes the transition to the R-phase favored, and there is therefore a temperature at the beginning of the transformation to R, i.e., R_s_, which is higher than the temperature at the beginning of the transformation to B19′, i.e., M_s_ [10,11].

If we have an endodontic file in the austenitic phase and we apply stress when introducing the file into the root canal, the austenite of the NiTi will be transformed into martensite induced by stress, exceeding levels of deformation much higher (up to 15%) compared to conventional metals [12,13]. When the clinician removes the file from the canal, and in the absence of stress, the NiTi will recover the austenite phase; i.e., a retransformation of the stress induced martensite takes place. This great elastic and flexibility behavior and its adaptation to the canal make NiTi an alloy for the application of this metal in endodontic files [14,15]. The transformation temperatures are the key to the properties and these depend fundamentally on the chemical composition. One of the biggest problems for NiTi instrumentation manufacturers is the reproducibility of the chemical composition. Normally, the alloys are equiatomic or very close to 50% atomic composition of each other, small variations in the contents of nickel or titanium will vary the transformation temperatures and therefore the phases of the alloy. Also grain sizes and dislocation density can affect transformation temperatures but not as strongly as variations in chemical composition [16,17].

Fractures that occur in endodontic files are fatigue and torsional fractures. The cracks appear in the areas of maximum stress that coincide with the point of maximum curvature in the dental canal. The fractures of NiTi files are due to the fact that the load cycles produce stress-induced martensitic transformations, and with the number of cycles, dislocations appear, and these can anchor the martensite plates and prevent them from re-transforming to austenite when the mechanical load is released [18]. These martensite plates are called stabilized martensite and cause the file to lose superelasticity and become an alloy without superelastic properties and consequently lose the extra toughness of superelastic alloys. It has been demonstrated for endodontic files that as the number of cycles of use increases, the enthalpies of transformation and retransformation are reduced until in the fracture cycle these energy values are practically zero. This decrease in enthalpies indicates that the energy to produce transformation and retransformation is decreasing because there is more and more stabilized martensite that, anchored by defects, is not susceptible to transformation [18,19,20]. 

The aim of this contribution is a laboratory study to determine the improvements in the fatigue behavior of alloys with the R-phase in their structure. The effect of the R-phase at different angles of application in the root canal will be studied with respect to files that do not present this R-phase. This study will make it possible to determine the advantages of this type of alloy with respect to conventional NiTi. In addition, the effects of the aging process, thermal cycling, and annealing process are analyzed. These aspects have to be considered by the clinicians to use the most suitable files with adequate treatments. 

## 2. Materials and Methods

### 2.1. Materials

Four NiTi endodontic rotary files were studied.

- Protaper Ultimate^TM^ F2 008v (Denstply Sirona, Ballaigues, Switzerland). Diameter 2.5 mm and length of working 16.0 mm. (Protaper)
- F6 SkyTaper^®^ files 25 mm. (Komet Lemgo, Nordrhein Westfalen, Germany). Diameter 2.5 mm and length of working 16.5 mm. (F6)
- Reciproc^®^. V040252025025. 25 mm, (VDW, Munchen, Germany). Diameter 2.5 mm and length of working 16.2 mm. (Reciproc)
- M-wire. Vortex Rotary Files. 25 mm (Dentsply Sirona, Johnson City, TN, USA). Diameter 2.5 mm and length of working 16.0 mm. (M-wire)

The chemical composition for Protaper was 49.5% in weight of nickel and 50.5% in weight of titanium, and for F6, it was 49.7% and 50.3% in weight of nickel and titanium, respectively. For Reciproc, the composition was 50.8% and 49.2% in weight of nickel and titanium, respectively, and for M-wire, 50.5% and 49.5 in weight of nickel and titanium, respectively. Other chemical elements were not detected. The chemical composition was measured by means of energy-dispersive X-ray microanalysis with a sensitivity of 0.1%.

Protaper and F6 are NiTi endodontic files with austenitic–martensite transformation. Reciproc and M-wire presented austenite with R-phase which transforms to martensite. The length of the four files studied was 25 mm in all cases. 

**Figure 1:**
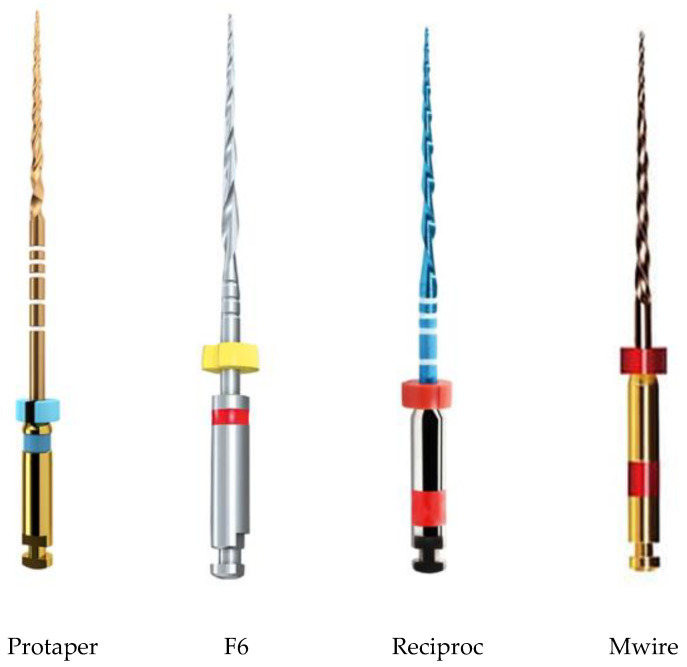
Different NiTi endodontic files studied. Reciproc and M wire present R-phase.

The endodontic files used are of the brand (Figure 1) and are placed on a motor X-Smart^®^ of Dentsply Sirona (Charlotte, NC, USA) with a speed of 300 rpm and torque of 2.2 N cm.

**Figure 2:**
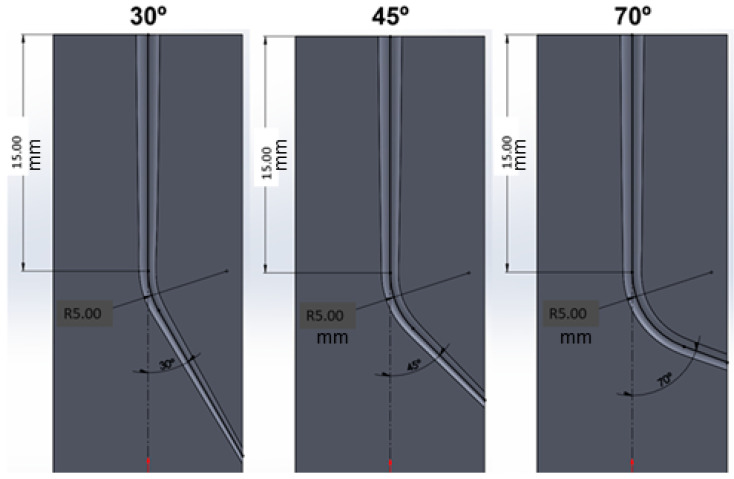
Scheme of the different molds for endodontics tests. The 30°, 45° and 70° angles were used, which are common angles in clinical practice [21].

Endodontic cycles are performed on molds made of polyamide with properties very similar to the natural tooth obtained by 3D printing with canals with angles of 30, 45 and 70°. These are the most common tooth canal angles in patients [21]. The file force on the mold was 5.5 N, and they were lubricated every 25 cycles (5 s) with an aqueous solution of 5% sodium hypochlorite. For the determination of the force to which the file is subjected on the mold, different measurements were performed by 25 clinicians of the Clínica Universitaria Odontológica Alfonso X el Sabio, obtaining a mean value of 5.5 N with a standard deviation of 2.8 N. The high precision dynamometer was Adamel Lombhragy (X1234, Lyon, France) and is adjusted to the hand of the clinician who will perform the tests with maximum force control adjusted. The diagrams of the molds used can be seen in Figure 2. 

For the test, it was important that the lubrication of the file was as similar as possible to what occurs in the clinic, and for this purpose, the instrument was soaked in the aqueous solution, which was maintained at 37 °C. This meant that the variations in the temperature of the file were negligible, as this could affect the phases present in the file if they were overheated.

Cycles to fracture were made for each type of endodontic files. The fracture files were also observed by scanning electron microscopy using a JEOL 6400 (JEOL, Tokyo, Japan) and transmission electron microscopy was performed using a JEOL 1200 EXII. Microscopy coupled with energy-dispersive X-ray microanalysis (Oxford Instruments, Oxford, UK) was used for determining the chemical composition.

The number of endodontic files used was analyzed by experiment design. The total number was 5 files for each endodontic file × 4 types of files × 3 angles studied = 60 samples.

### 2.2. Calorimetric Tests

Five samples for each endodontic rotary file and for each thermal treatment were analyzed, all of them 25.0 mm long and 0.46 mm in diameter. The transformation temperatures were measured by means of a calorimeter Melcor S 10. The calorimetric system used was based on a flow calorimeter, which measured differential signals (ΔT) by means of thermocouples batteries. The temperature was measured by means of a standard Pt-100 probe. All signals were digitalized through a multichannel recorder and linked to a microcomputer [20]. All signals were digitalized through a multichannel recorder and linked to a microcomputer. M_s_, R_s_ and A_s_ transformation temperatures occur when there is a sudden increment in calorimetric signal. In the same way, the final temperatures, M_f_, R_f_ and A_f_, are determined when the calorimetric signal returns to the baseline. The method used to determine the transformation and retransformation temperatures was the tangent [21,22].

### 2.3. Mechanical Cycles

Mechanical cycling was carried out at the 30, 45 and 70° angles. Ten files of each type and for each angle were cycled at different number of cycles to fracture. The files were cooled, as indicated above, in order to simulate clinical reality as closely as possible. For the different cycles, the transformation temperatures M_s_ and A_f_ were determined calorimetrically for the Protaper and F6 files, and for the Reciproc and M-wire files, the temperatures Ms and A_f_ were determined; for the start and end temperatures of the R-phase transformation, R_s_ and R_f_ were determined for the files tested at 45°. Fractography was studied by SEM. 

### 2.4. Aging Treatment

The files were subjected to heat treatments of 300 and 500 °C for different times: 15, 30, 45, 60, 90 and 120 min and after the files were quenched in water at a temperature of 25 °C. Six files of each system were treated at the different temperatures and times, and the transformation temperatures M_s_ and A_f_ were studied for all the samples and R_s_ and R_f_ were studied for the Reciproc and M-wire.

### 2.5. Preparation of the Samples for the Observation by Transmission Electron Microscopy (TEM)

The process of focused ion beam (FIB) cutting was carried out using a CrossBeam (1560XB, Carl Zeiss, Berlin, Germany). A 1 μm thick platinum strip was deposited on the top of the area of interest to protect the surface from ion beam induced damage and unwanted milling during sample preparation. Two trenches were cut by using the Ga FIB (first at a current of 10 nA and finally at 2 nA), one from each side, leaving behind a thin electron-transparent lamella supported by bulk material on two opposite sides. The specimen was attached to the micromanipulator tip via platinum deposition. After that, the lamella was removed from its trench. Further FIB thinning at a lower beam current (first 500 pA, then 200 pA, 100 pA and finally 50 pA) was performed on both sides of the wall, leading to a final membrane thickness of 50–100 nm. Finally, the lamella was transferred to a TEM grid and attached by depositing a platinum layer. TEM used was (JEOL, F1400, Tokyo, Japan) using 120 KV.

### 2.6. Statistical Analysis

The data were statistically analyzed using Student’s *t*-tests, one-way ANOVA tables and Tukey’s multiple comparison tests in order to evaluate any statistically significant differences between the sample groups. The differences were considered significant when *p* < 0.01. All statistical analyses were performed with MinitabTM software (Minitab release 13.0, Minitab Inc., State College, PA, USA).

## 3. Results

**Figure 3:**
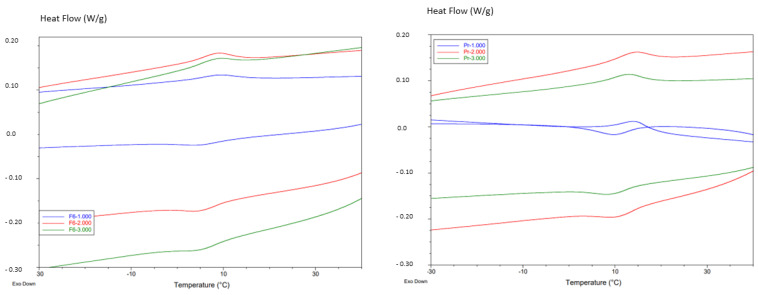
Thermograms of F6 and Protaper endodontic files (heating and cooling cycles). Three different samples studied for each type of files.

**Figure 4:**
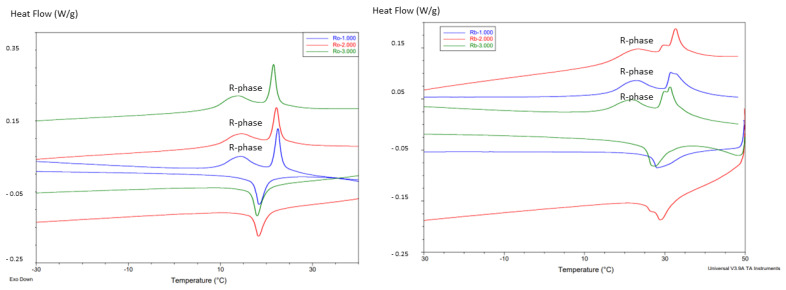
Thermograms of M-wire and Reciproc endodontic files (heating and cooling cycles). Three different samples studied for each type of files. In the thermograms the peaks of R-phase are identified.

The transformation temperatures of the four types of endodontic files are shown in Table 1. In all cases, at oral temperature, the files present for the Protaper and F6 cases a single peak showing the transformation of the austenitic to martensitic phase. However, in the thermograms of the Reciproc and M-wire files there is a double peak corresponding to the appearance of the R-phase in these files. Figure 3 shows the thermograms of Protaper and F6, and Figure 4 shows the thermograms of the endodontic files corresponding to Reciproc and M-wire. The transformation temperatures are shown in Table 1. 

**Figure 5:**
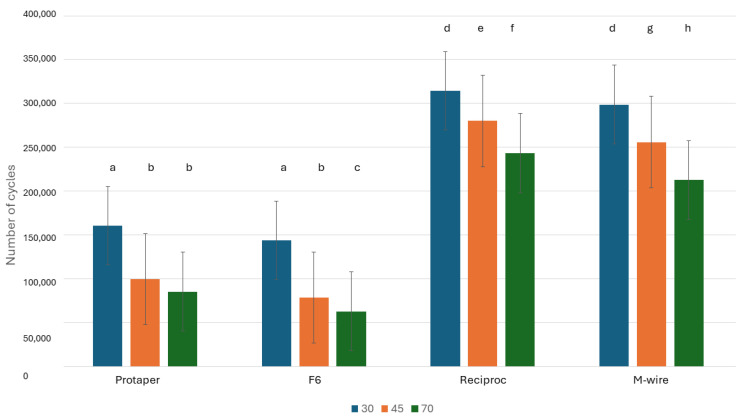
Number of cycles to fracture for different types of files and different angles. Letters signify statistically significant differences with a *p* < 0.01 with respect to the number of cycles to fracture. When the letters are equal there are no statistically significant differences between them. If the letters are different, it means that there are statistically significant differences with a *p* < 0.01.

The results of the number of cycles to fracture for each type of file at different angles of use can be observed in Figure 5. For each angle we can observe statistically significant differences between the values of Protaper and F6 with respect to the endodontic files containing R-Phase. Reciproc and M-wire values were *p* < 0.01.

It can be observed for all the files that the number of cycles decreases as the degree of inclination of the endodontic file increases. This fact is due to the higher bending moment of the file. Likewise, it can be seen how the number of cycles of the Reciproc and M-wire files that present the R-phase fracture at a higher number of cycles than the Protaper and F6 files that do not present the R-phase in their microstructure.

**Figure 6:**
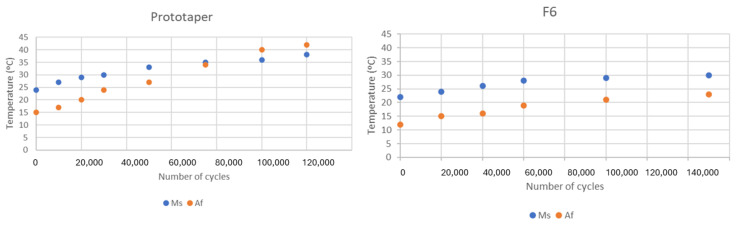
M_s_ and A_f_ transformation temperatures at different number of cycles for test realized at 45° with Protaper and F6 files.

**Figure 7:**
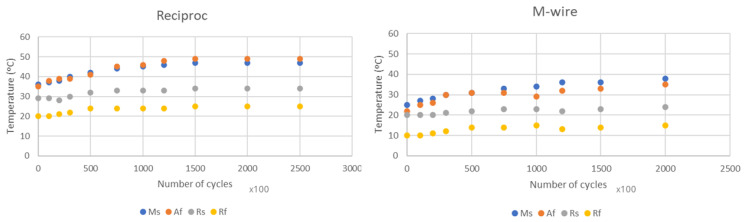
M_s_ and A_f_ transformation temperatures and R_s_ and R_f_ for the R transition at different number of cycles for test realized at 45° with Reciproc and M-wire files.

Calorimetric studies were carried out at different cycles of the different milling files to determine the transformation temperatures at the beginning of the martensitic transformation (M_s_) and the temperature at the end of the retransformation to austenite (A_f_), which indicates how easy it is to cause the appearance of martensite and the temperature necessary to cause the return to the austenitic phase. For the Reciproc and M-wire files, the transformation temperatures for the initiation of the transformation from austenite to R-phase were determined, as well as those for the retransformation from the R-phase to the fully austenitic structure. Figure 6 corresponds to the M_s_ and A_f_ temperatures for the files without the R-phase and Figure 7 for the samples showing the intermediate transformation to the R-phase.

**Figure 8:**
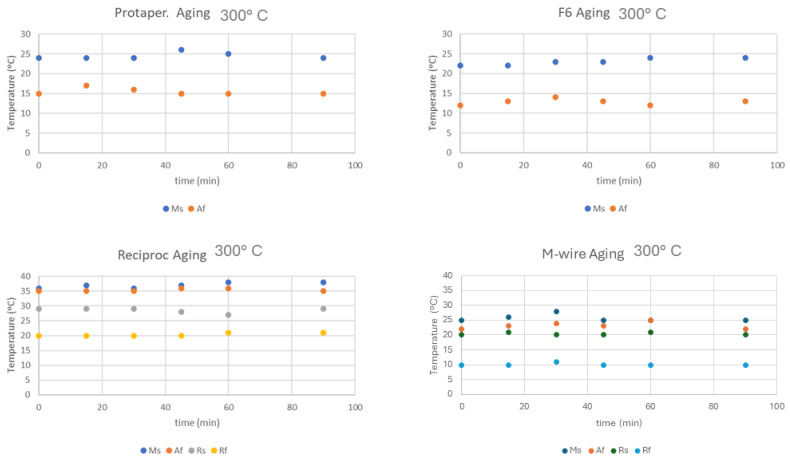
Transformation temperatures for each type of file treated at 300 °C for different times.

Figure 8 shows the results of the transformation temperatures for the four types of files at a temperature of 300 °C during different dwell times. As can be seen, in no case are significant changes seen in any of the transformation temperatures determined.

**Figure 9:**
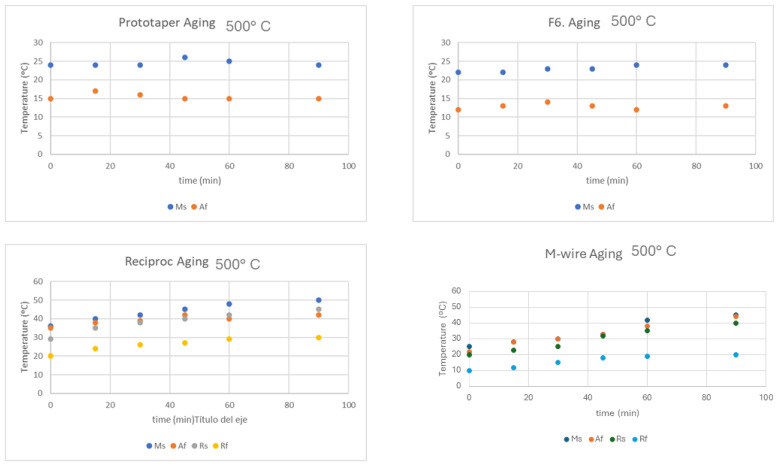
Transformation temperatures for each type of file treated at 500 °C for different times.

Figure 9 shows the transformation temperatures at different times when the aging temperature is 500 °C. In the latter case, it can be seen that the transformation temperatures for the milling cutters with the presence of the R-phase show an increase in transformation temperatures, which means that the formation of the martensitic phase and the transition to the R-phase is favored, and the increases in A_f_ show that it is necessary to further increase the temperature to cause the transformation from martensite to austenite.

**Figure 10:**
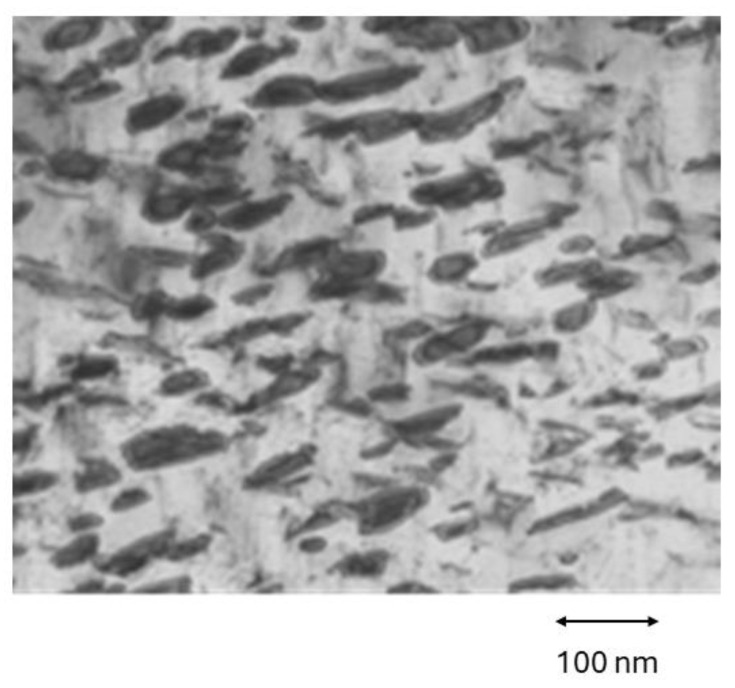
Precipitates observed by Transmission Electron Microscope produced by aging in Ni-rich samples.

It has been observed by Transmission Electron Microscopy the effects of thermal aging treatments for nickel-rich files that produce the appearance of precipitates Ni-rich that causes a variation in the chemical composition of the matrix causing the variation of the properties. The precipitates observed in the R-phase samples for the 50.8 wt.% files can be seen in Figure 10 with an elongated morphology [23,24,25].

**Figure 11:**
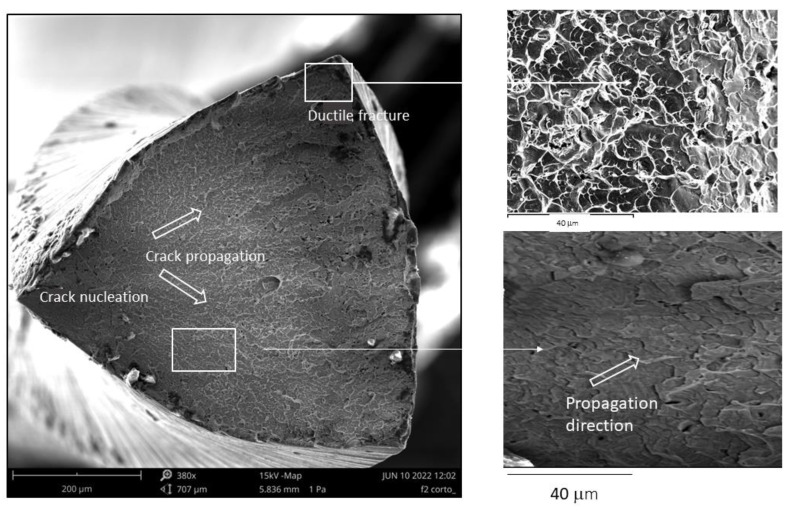
Fractography of the NiTi endodontic file. In this case, it corresponds to the M-wire.

Fractography studies were carried out by scanning electron microcopy on the endodontic files and in all cases the fractures due to fatigue were observed. Figure 11 shows the fractography, observing the crack nucleation zone, propagation and ductile fracture.

## 4. Discussion

The endodontic files studied have very similar dimensions and designs, which will allow us to determine the influence of the R phase on the fatigue behavior at different angles. The chemical compositions for Protaper were 49.5% and for F6 49.7% in weight of nickel. The files with R phase present a higher content of nickel, 50.8 and 50.5 % in weight of nickel for Reciproc and M-wire, respectively. In all cases, the phase present is austenite, which ensures the superelastic behavior of the files. However, the Reciproc and M-wire files have been shown to exhibit the R-phase. 

As is well known, at body temperature (37 °C) the endodontic files are in austenitic phase (T > M_s_), i.e., they present the possibility to produce stress-induced martensitic transformation. That is, when the endodontic file is flexed in the stress zones, the austenite transforms to stress-induced martensite, which gives it flexibility. If the temperature is lower than the temperature M_f_, temperature at which the whole alloy is martensite, the endodontic file will present the shape memory property and can be easily deformed. However, the endodontic file does not have the ability as in the case of austenite to absorb energy for stress-induced martensitic transformation [26,27]. The flexibility of the file is due to the possibility that the martensite plates are oriented in the direction of mechanical stress. These martensite plates can orient themselves and even grow at each other’s expense, with the plates with better crystalline orientation increasing in size with respect to the disfavored ones. This self-accommodation mechanism allows energy absorption and is due to the thermoelasticity of the transformation [28,29].

The experimental results of this study are in agreement with the investigations of Prati et al. [30,31]. These researchers obtained a numerical simulation using the finite element method of the stresses produced in dental canals by NiTi endodontic files. In this work it was observed that the highest stress values were produced in healthy teeth and with a good mineralization, it was observed that the conicity between values of 4 and 6% did not produce statistically significant differences. It was also found that an important factor in the von Mises stress and strain values corresponded to the modulus of elasticity of the files. Therefore, the presence of the R-phase modifying the elastic behavior of the files produces important changes as we have seen in this research. The different designs studied by these authors showed that all files produced a concentration of stresses in the curvature of the root canal area approximately 7 mm from the apex. 

As can be seen from the results in Figure 5, the fatigue cycle values of the R-phase files are much higher, with statistically significant differences (*p* < 0.01) with respect to those without R-phase.

This work validates the finite element study of Santos et al. [32] It can be seen that the endodontic files with the lowest bending values are the microstructures with R-phase. The two-phase structures with austenite and R-phase increase bending and the one that presents higher bending values is when the microstructure presents the austenitic structure. Consequently, the R-phase files are more flexible without affecting their rotational stress behavior. These results obtained by the finite element method are validated with our results where it has been verified that the presence of R phase behaves better in fatigue without loss of bending requirements.

It can be observed in both types of files that the mechanical cycles cause an increase in the transformation temperatures M_s_ and A_f_, i.e., the martensite phase increases the stability range. Firstly, the mechanical cycles create lattice defects that have an internal energy associated with them. This energy helps the martensitic transformation in the same way that it has been observed that the fine grain size causes an increase in the M_s_ temperature due to the energy of the grain boundary [33,34]. The explanation for the temperature A_f_ is due to the anchoring of the martensite plates in the defects produced by the mechanical cycles. The plates are stabilized in the defects and an increase in heating temperatures is necessary to unblock the plates and allow them to re-transform to the austenitic phase. Sometimes, heating is not sufficient to return to the austenitic phase due to the stability of the martensite. When this happens, we can only return to austenite by increasing the temperature to the austenization values of the equilibrium diagram above 850 °C [35]. Stressing the material with stabilized martensite will cause it to break, as it has no more energy absorption mechanisms. After the elastic behavior of the martensite, its orientation and the coalescence of plates, NiTi fractures. It can also be seen that in the Reciproc and M-wire files the temperatures R_s_ and R_f_ do not vary as much as M_s_ and A_f_ due to the low energy dispersion in the R-transition [36,37,38,39]. This can be corroborated by the small hysteresis of the R-transitions observed in the calorimetry compared to the B19’ transformations. The same effect has been reported for the stress-induced transformations in the pseudoelastic curves obtained. 

NiTi ageing processes occur in Ni-rich alloys, forming coherent Ni_4_Ti_3_ precipitates that cause the nickel content of the matrix phase to decrease, leading to concentration gradients around the precipitates [40,41,42]. The decrease of nickel increases the transformation temperatures R_s_. In the same way, the formation of the precipitates generates a deformation field generated by the coherent precipitates that favor the formation of the R-phase. However, it is the role of the chemical composition gradient that most strongly affects the increase of the transformation temperatures R_s_. 

From Figure 9, it can be seen that for the 4 files studied for ageing at 300 °C, no changes can be observed in the transformation temperatures and, therefore, there is no formation of precipitates. However, it can be seen that at a temperature of 500 °C, in the files rich in nickel content, which are those with R phase, there is an increase in the R_s_ and R_f_ temperatures, which indicates the appearance of precipitates. As the treatment time increases, the number of precipitates increases and therefore the nickel impoverishment in the provoke matrix increases by almost 20 °C in the R_s_ temperature for times from 120 °C to a temperature of 500 °C. Some authors describe that precipitates can appear from treatments as low as 250 °C, with very fine and coherent precipitates appearing. However, possible changes in transformation temperatures occur at longer residence times (50 h), and here we have only studied a time period of up to 2 h [43,44].

As is well known, in general, endodontic files do not exceed temperatures of 130 °C due to autoclave sterilization processes. In some cases, it has been possible to exceed these values in the mouth, but they are punctual cases due to unusual friction, but they last a very short time. In this research, we wanted to see the limits of the aging of NiTi alloys used in endodontics and to demonstrate that there is no possible change of properties with the usual temperatures in the use of endodontic files. However, sometimes, some orthodontists and endodontists perform thermal annealing treatments to eliminate dislocations and stabilize martensite plates that impair the superelastic behavior of wires or files. These are heat treatments, where temperatures can range from 500 to 800 °C, that usually last about 15 min and require the subsequent quenching in water [44,45]. In these cases, these treatments must be taken into account.

One of the aspects to take into consideration is that during the mechanical and chemical preparation of root canals with rotary endodontic files, an accidental extrusion of debris beyond the apex can occur. The term flare-up is commonly used to indicate a clinical condition in which pain and swelling of the oral mucosa and soft facial tissues are observed following root canal therapy of a dental element. The work of Villani et al. [46] found that the apical extrusion of debris occurs both with the reciprocating technique with continuously rotating instruments. The association between mucosal inflammation and pain and debris extrusion during the shaping phase needs to be demonstrated with in vivo clinical investigations.

One of the limitations of this study is that only rotational and not reciprocating NiTi files have been performed. One of the future studies could be the realization of the same study in this other type of files. One of the aspects that clinicians should take into account in endodontic therapy is the use of files with rotational or reciprocating movement. Nouri et al. [47] found that there is no significant difference in the penetration of root canals as long as they are straight or moderately curved. For more pronounced curvatures, it has been found that reciprocating files tend to perform better but the results are not conclusive. The differences cannot be attributed to the microstructure of the endodontic file but to the stress distribution caused by the reciprocal movement and the better adaptation to the canal reaching the apical forament as well as the better distribution of loads with respect to the axis of rotation (back-and-forth rotation) [48].

Fractography shows that it is produced by fatigue-torsion as usual in endodontic files, observing that the final part of the fracture is ductile, which indicates a good toughness of the material. No brittle fracture was observed in any area of the fracture surface. Undoubtedly, the presence of precipitates will cause a change in the fracture behavior of the file, decreasing the ductility. Another factor to be taken into account in the behavior of NiTi endodontic files is the physicochemical properties of the surface as well as the variation of the microstructures due to the effect of heat treatments. This aspect was studied by Azizi et al. [49] where they compared conventional (OShape) and heat-treated (OCurve) NiTi endodontic files. The results illustrated that heat treatments increase the fatigue life (*p* < 0.05) compared to conventional ones. The authors point out that the formation of a TiO_2_ layer causes compressive states on the surface and causes a delay in crack nucleation. Another interesting aspect is the influence of the sterilization process where the autoclave causes an increase of martensite in the heat-treated files. The results of the studies of Azizi at al. [49] conclude that heat treatments improve the mechanical behavior of the files as well as a better adaptation to the root canal and have opened a field of research for the optimization of heat treatments in order to obtain more flexible files with better mechanical behavior in the long term.

## 5. Conclusions

Increased insertion angles in canals reduce the service time of endodontic files. NiTi files with R-phase presence improve the durability of the files due to the additional transformation from B2 to R-phase and from R-phase to B19’. This increase in absorbed energy (toughness) leads to better fatigue behavior of R-phase files. Clinicians should note that mechanical cycling affects the properties of the files by increasing the transformation temperatures M_s_ and A_f_ which favors transformation as well as hinders retransformation. For files with the presence of R phase, it has been found that the temperatures R_s_ and R_f_ increase less significantly due to the small hysteresis of the transition to the R phase. The aging processes only affect NiTi files when heat treatments are performed at 500 °C due to the appearance of Ni_4_Ti_3_ precipitates for the alloys presenting R transition because they are nickel-rich alloys. Therefore, clinicians should avoid annealing heat treatments to improve superelasticity above 500 °C as NiTi file loses properties. These results can help clinicians understand its properties and improve the use of endodontic files.

## Figures and Tables

**Table 1:** Transformation and retransformation temperatures in °C for different endodontic files.

Endodontic File	M_s_	M_f_	A_s_	A_f_	R_s_	R_f_
Protaper	24 ± 3	15 ± 1	3 ± 4	34 ± 3	-	-
F6	22 ± 4	12 ± 2	−4 ± 3	30 ± 2	-	-
Reciproc	36 ± 5	35 ± 3	25 ± 5	40 ± 7	20 ± 2	29 ± 4
M-wire	25 ± 4	22 ± 2	15 ± 4	29 ± 6	10 ± 3	18 ± 3

## Data Availability

The authors can provide details of the research upon request by letter and commenting on their needs.
